# The Minimum Effective Concentration (MEC95) of different volumes of ropivacaine for ultrasound-guided caudal epidural block: a dose-finding study

**DOI:** 10.1186/s12871-023-02026-y

**Published:** 2023-03-09

**Authors:** Dongmei Ma, Yan Chen, Ping Chen, Jianhong Xu, Jian Guo, Lijia Peng

**Affiliations:** 1 Department of Anesthesiology, The Fourth Affiliated Hospital, Zhejiang University School of Medicine, Zhejiang, China; 2 Department of Anesthesiology, The Sixth Clinical Medical College of Xinjiang Medical University, Urumqi, Xinjiang China

**Keywords:** Caudal epidural block, Ultrasound, Dixon up-and-down method

## Abstract

**Background:**

Caudal epidural block (CEB) may be beneficial in anorectal surgery because its use may extend postoperative analgesia. This dose-finding study aimed to estimate the minimum effective anesthetic concentrations for 95% patients(MEC95) of 20 ml or 25 ml of ropivacaine in with CEB.

**Patients and methods:**

In this double-blind, prospective study, the concentration of ropivacaine administered in 20 ml and 25 ml for ultrasound-guided CEB were determined using the sample up-and-down sequential allocation study design of binary response variables. The first participant was given 0.5% ropivacaine. Depending on whether a block was successful or unsuccessful, the concentration of local anesthesia was decreased or increased by 0.025% in the next patient. Every five minutes for 30 min, the sensory blockade using a pin-prick sensation at S3 dermatome compared to at T6 dermatome were evaluated every 5 min within 30 min. An effective CEB was defined as a a reduction of sensation at S3 dermatome and the existence of flaccid anal sphincter. Anesthesia was considered successful if the surgeon could perform the surgery without additional anesthesia. We determined the MEC50 using the Dixon and Massey up-and-down method and estimated the MEC95 using probit regression.

**Results:**

The concentration of ropivacaine administered in 20 ml for CEB ranged from 0.2% to 0.5%. Probit regression with a bias-corrected Morris 95% CI derived by bootstrapping showed an MEC50 and MEC 50 of ropivacaine for anorectal surgical anesthesia were 0.27% (95% CI, 0.24 to 0.31) and 0.36%(95% CI, 0.32 to 0.61). The concentration of ropivacaine administered in 25 ml for CEB ranged from 0.175 to 0.5. Probit regression with a bias-corrected Morris 95% CI derived by bootstrapping showed an MEC50 and MEC95 for CEB were 0.24% (95% CI, 0.19 to 0.27) and 0.32% (95% CI, 0.28 to 0.54).

**Conclusion:**

With ultrasound-guided CEB, the MEC95 of 0.36% ropivacaine at 20 ml and 0.32% ropivacaine at 25 ml provide adequate surgical anesthesia/analgesia 95% of patients undergoing anorectoal surgery.

**Trial registration:**

Clinicaltrails.gov: Retrospectively registered (ChiCTR2100042954; Registration date:1/2, 2021).

## Introduction

Caudal epidural block (CEB) is a kind of epidural anesthesia in which local anesthesia is injected through the sacral hiatus into the sacral epidural space to block the sacral nerve and temporarily paralyze the area it controls [1]. CEB may be beneficial in anorectal surgery because its use may extend postoperative analgesia, which reduce the need for systemic analgesics and their potential side effects [2, 3]. Although CEB has many clinical applications, it is sometimes difficult to ascertain the anatomical location of the sacral hiatus and the caudal epidural space, particularly in adults [4, 5]. Considering the many anatomical variations of the sacral hiatus and cornua sacralia, the success rate of the classic caudal epidural anesthesia method in adult patients is approximately 68–75% [5], and in children, the blind technique has a success rate of over 96% [6]. In addition, due to the abundance of the sacral venous plexus, local anesthesia poisoning reactions can easily occur [2]. These factors restrict the use of CEB in adults.

Ultrasonography has been reported to be beneficial in administering regional anesthesia. This technique is useful for visualizing the sacral hiatus and sacrococcygeal ligament, inserting the needle, and distributing the local anesthetic agent. Klocke and colleagues described ultrasound-guided CEB in 2003, and it has become increasingly popular [7]. Various studies have reported very high success rates (96.9%-100%) for ultrasound-guided CEB in diverse ethnic groups [8, 9]. The advantages of ultrasound-guided CEB are simplicity of operator, minimal trauma, few complications and obvious analgesic effects.

In clinical, minor anorectal surgeries are often performed in day care facilities, using spinal anesthesia modified into a saddle block. However, CEB offers certain advantages. such as minimal motor blockade, early mobilization, lesser degree of hypotension, and lesser chances of postoperative puncture headache. Other potential advantages of the caudal route include ease of positioning and selective blocking of sacral nerves. Ultrasound-guided CEB can be considered as an option for anorectal procedures of short duration with acceptable success rates, surgical conditions, and patient comfort. [9, 10], in addition, high doses of local anesthetics increase the risk of complications and toxicity. The minimal effective concentration [11, 12] or minimal effective volume [13–15] are commonly used to minimize the required local anesthetic dose [16]. It is common to use the up-and-down sequential method to estimate the effective concentration or volume for 50% (MEC50 or MEV50), but both are of limited clinical significance, which, by definition, indicates that they have a 50% failure rate. Hence, there is a need to identify the minimal effective concentration or volume of ropivacaine in 95% of patients to produce a successful CEB. The purpose of this study was to evaluate the effective concentration of ropivacaine during ultrasound-guided CEB for administration in 20 ml or 25 ml in 95% of patients.

## Material and methods

This single-armed prospective study was approved by the Ethics Committee of the Fourth Affiliated Hospital of Zhejiang University Medical College (YiWu, People’s Republic of China) (No: 202–031). The trial was registered with the Clinical Trial Registry in the 01/02/2021(No: ChiCTR2100042954). The period of enrollment was from March 2021 to February 2022. A total of 80 patients who were scheduled for elective anal fistulectomy or hemorrhoidectomy were selected. The inclusion criteria were (1) age between 18 and 65 years; (2) American Society of Anesthesiologists (ASA) physical status I to II; and (3) body mass index between 18 and 35 kg/m^2^. The exclusion criteria were as follows: (1) local infection in the patient’s caudal region, (2) hypersensitivity to amide local anesthetics, and (3) preexisting neuralgic or obvious spinal disease.

An 18- or 20-gauge intravenous catheter was inserted into the upper limb in the induction room. During the procedure, noninvasive systemic blood pressure and heart rate were measured using an automatic cycling device (Cardiocap; Mindray). Each block was administered by one experienced anesthetist using an S-Nerve device (SonoSite Inc.). During block placement, no analgesia or sedation was given to the patients.

All eligible patients were randomly divided, using a random number table, into one of two groups; group 1 received 20 ml (*n =* 39) while, group 2 received 25 ml (*n =* 37) of ropivacaine. All patients received US-guided CEB in the prone position in the induction room. Under sterile conditions, the short axis of the ultrasonic probe was placed in transverse orientation to identify the bilateral sacral horn, sacral caudal ligament and sacrum, and to locate the sacral hiatus– appearing like a frog’s face. Then, the ultrasonic rotation 90-degree long axis was placed to obtain the longitudinal view of the sacral hiatus, sacral caudal ligament and sacrum. A 20-G needle was inserted into the sacral hiatus through the sacrococcygeal ligament. Caudal space was identified using ultrasound-guided in-plane puncture technique, which allowed me to see the needle's entire length, as well as loss of resistance technique using saline. The needle then was further gently advanced 0.5 cm to avoid spinal puncture. After negative aspiration,1 ml of a solution containing 5ug epinephrine was administrated as a test dose. If after 1 min there was no evidence of intravascular injection and then a prepared-20 mL (group 1) or -25 mL (group 2) of ropivacaine (Naropin; AstraZeneca, Sodertalje, Sweden) was injected. A prepared-CEB drugs were diluted with 0.9% saline to achieve the desired concentration without epinephrine, which lasted for 2 min.

During the ultrasound-guided CEB, the block performance time (defined as the time from the contact of the ultrasound probe with the patient to the end of the local anesthetic injection) was recorded. In this study, the effectiveness of CEB was assessed through pinprick tests of the perineal area and a lax anal sphincter laxity test was performed after local anesthetics were administered in five-minute intervals for 30 min after. After the local anesthetic was injected, researcher checked the sensations of pinprick test every five minutes in the S3 and T6 dermatome areas at every five minutes for 30 min by a blinded observer. Motor block was evaluated at the end of surgery according to the Bromage scale (0 = full flexion of feet and knees, 1 = just able to move knees, 2 = able to move feet only, and 3 = unable to move feet or knees) performed at 5, 10, 15 min following administration of ropivacaine and at the end of the surgery. In the study, the pinprick test was used to determine the block onset time (defined as the time interval between the end of the local anesthetic injection to less pain in the S3 dermatome areas than T6 dermatome areas) and the block perfect time (defined as the time interval between the end of the local anesthetic injection to a painless pinprick in the S3 dermatome areas and the existence of flaccid anal sphincter) [17]. Any adverse events were recorded, such as nausea, vomiting, local anesthetic systemic toxicity, vascular puncture, residual block, and persistent neurologic deficit. As a rule, hypotension is defined as a 20% decrease in systolic blood pressure compared to the preanesthetic value or 90 mmHg of systolic blood pressure. Hypotension was treated with 6 mg of ephedrine intravenously and crystalloid fluids. Bradycardia (50 bpm) was treated with 0.5 mg of atropine intravenously.

The block was considered effective if the patient's anal sphincter laxed 30 min after the local anesthetic was injected, and pain-free surgery was achieved without the need for rescue blocks including supplemental opioids, general anesthesia or local infiltration by the surgeon. The block was considered ineffective if there was pain during surgery or the presence of a tight anal sphincter, and the patient received rescue blocks including supplemental opioids, general anesthesia or local infiltration by the surgeon.

For postoperative, patients were assessed pain score using the numerical rating scale (NRS; 0 = no pain; 10 = most severe pain imaginable), motor block, numbness, and complications or side effects after operation by an anesthesiologist. Patients were prescribed ketorolac 30 mg intravenously up to 3 times a day if NRS scores were higher than 3. The duration of analgesia, defined as the interval between the block onset time and the initial use of rescue analgesia for surgical site pain.

The current study was a double-blind design because the individuals involved in collecting and analyzing data were unaware of the group assignment. The assessment was performed by an independent outcome assessor who was blind to the concentration of local anesthetic that was injected and did not know the result of previously case.

### Statistical analysis

The primary goal of this dose-finding study was to estimate the MEC95 for US-guided CEB. Through a small-sample up-and-down sequential study design of binary allocation response variables as described by Saranteas et al. [18], we determined the concentration of local anesthetic (20 ml and25ml of ropivacaine) to be administered through the efficiency of block. The first patients in both groups received a concentration of 0.5% (20 ml in group 1 and 25 ml in group 2) of ropivacaine. The next case concentration of ropivacaine was determined by the effectiveness of CEB from previous case. According to Dixon’s up-and-down sequential method, ineffective block resulted in a 0.025% increase in the ropivacaine concentration of the next patient, while effective block resulted in a 0.025% decrease ropivacaine concentration.

Dixon and Massey determined the sample size by using the formula, *n =* 2(SD/SEM)^2^[19]. With a 0.05 SD and 0.012 SEM, 34 patients must be included in this study. Considering an attrition rate of 10%, we included 37 patients in each group. The MEC95 was estimated using probit regression. The data were processed using IBM SPSS Statistics version 23.0 (IBM Corporation, Armonk, New York). Mean (SD) values were analyzed by using the unpaired Student t test or Welch t test for different variances, median (interquartile) by using the Mann–Whitney U test. Categorical variables were reported as Number (proportion) and evaluated using Fisher’s exact or the X2 test where appropriate. For all tests, *P* < 0.05 was defined as statistically significant.

## Results

**Fig. 1:**
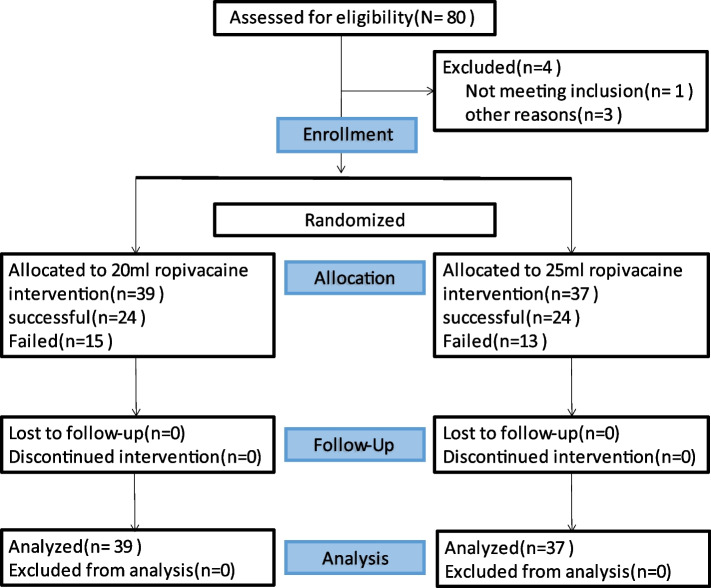
Participant flow diagram. A total of 80 patients were assessed for eligibility. 4 patients were excluded for some reasons. A total of 76 patients finally completed the protocol

Between March, 2021, and February, 2022, 80 patients were screened for inclusion, and 76 patients fulfilled the inclusion criteria and were enrolled. A participant flow diagram is showed in Fig. 1.

The characteristics of patients and clinical results are displayed in Table 1, and patients’ general characteristics, including age, weight, height, BMI, ASA score and types of surgery, between the two groups were comparable. The block performance time and onset time was about 4 min in both groups. The block perfect time of 25 ml group was a few longer than that of 20 ml group(*p >* 0.05). The block duration in the 25 ml group was about 435 min longer than in the 20 ml group (*p >* 0.05).

**Table 1:** Patient Characteristics and clinical results (*n =* 76)

	**20 ml of ropivacaine Group (*n =* 39)**	**25 ml of ropivacaine Group (*n =* 37)**	***P*-value**
Age, mean (SD),y	39.33(14)	39.89(11)	0.86
Sex (male/female), n	23/16	24/13	0.30
Weight, mean (SD), kg	65.14(10.82)	67.65(11.22)	0.33
Height, mean (SD), cm	165.92(8.13)	167.35(7.96)	0.43
BMI, mean (SD), kg/m^2^	23.61(3.26)	24.15(3.11)	0.24
ASA physical status (I/II), n	27/12	24/13	0.93
Types of surgery, n			0.95
Hemorrhoids	16	19	
Perianal abscess	8	8	
Anal fistula	8	5	
Anal polyp	7	5	
The block performance time, min	4.31(0.85)	4.32(1.18)	0.94
The block onset time, min	3.67(1.68)	3.65(2.19)	0.97
The block perfect time, min	9.57(3.26)	11.24(3.83)	0.11
The block duration time, min	409.92(74.15)	434.84(73.86)	0.27

Notes: Continuous variables and clinical results are presented as mean and standard deviation (SD); categorical variables are presented as counts

*BMI* Body mass index, *ASA* American society of anesthesiologists (physical status)

**Fig. 2:**
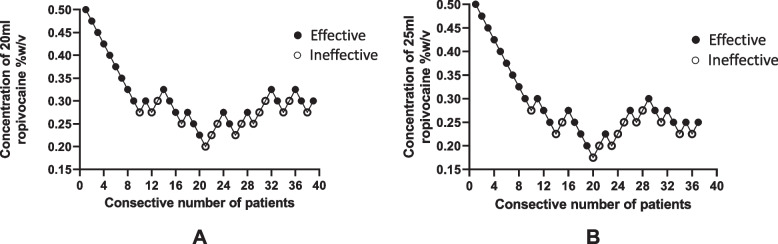
Sequential block results of ultrasound-guided sacral caudal epidural block using ropivacaine 20 ml (**A**) and 25 ml(**B**) according to Dixon and Massey up-and-down method

The up-and-down oscillation curves are illustrated in Fig. 2. The concentration of local anesthetic administered ranged from 0.175% to 0.5%. According to up-and-down method, the MEC50 was 0.276% (95% CI 0.236 to 0.308%) for 20 ml ropivacaine and 0.241% (95% CI 0.194 to 0.268%) for 25 ml ropivacaine. Then, using the probit regression model, the MEC95 was 0.362% (95% CI 0.322 to 0.612%) for 20 ml ropivacaine and 0.316% (95% CI 0.282 to 0.542%) for 25 ml ropivacaine. The Dixon up-and-down plots for each group are shown in Fig. 2A and B.

**Fig. 3:**
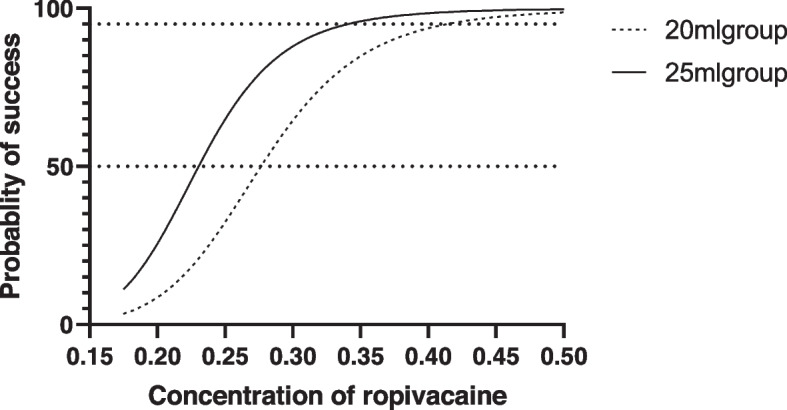
The dose–response curve for the MEC50 and MEC95 of ropivacaine in two groups. The MEC50 and MEC95 was 0.276% (95% CI 0.236 to 0.308%) and 0.362% (95% CI 0.322 to 0.612%) for 20 ml ropivacaine; The MEC50 and MEC95 was 0.241% (95% CI 0.194 to 0.268%) and 0.316% (95% CI 0.282 to 0.542%) for 25 ml ropivacaine

The dose–response curve for the MEC50 and MEC95 of ropivacaine in two groups (Fig. 3).

## Discussion

In this prospective, double-blind up-and-down sequential allocation study, we estimated the minimum effective concentration for 50%(MEC50) and 95%(MEC95) patients in two different volumes of ropivacaine for CEB. Ropivacaine is an amide-type local anesthetic with a long-acting mechanism of action that is usually well tolerated and considered to have a longer blocking effect, much has a lower cardiotoxicity and greater sensory and motor block separation than other local anesthetics. it has been widely used for CEB in children and adults [20–22]. Ropivacaine concentration in CEB ranges from 0.2–0.5%, have been used to administer a successful CEB without a significant influence on surgical anesthesia, thus indicating that local anesthetic concentrations at lower levels could also be effective [23–25]. In this dose-finding study, we chose 0.5% ropivacaine as the initial concentration to avoid deficient anesthesia and provide a reliable concentration. During this study, the effectiveness of CEB was evaluated by observing pain in the perineal area and relaxation of the anal sphincter. The block was considered ineffective if the patient suffered pain during the surgery, had a tight anal sphincter, or if oxycodone was administered intravenously or local anesthetics were injected. We found that the MEC50 for CEB of ropivacaine at 20 and 25 ml were 0.276% (95% CI 0.236 to 0.308%), and 0.241% (95% CI 0.194 to 0.268%) respectively. The calculated MEC95 for CEB of ropivacaine at 20 ml and 25 ml were 0.362% (95% CI 0.322 to 0.612%), and 0.316% (95% CI 0.282 to 0.542%) ropivacaine. The results were similar to a previous study by Li et al. [25], in which the authors found that the MEC50 of ropivacaine for caudal anesthesia was 0.296% in men and 0.389% in women with a fixed 20 ml volume by utilizing Dixon’s up-and-down sequential allocation. The study implies that the duration of CEB using a ropivacaine were 6–8 h (see Table 1), which was a longer analgesia effects than in Li et al.’s study [25]. This shows that CEB can produce good analgesic effects for anorectal surgery.

The choice of local anesthetic agent, doses, concentrations, and volume can influence the onset, spread, quality of blockade, and duration of anesthesia [26, 27]. There are a few studies reported that a local anesthetic volume is more important than a concentration for peripheral nerve blockade [27]. Literature notes that the adequate spreading for caudal anesthesia in adults is required 30-32 ml [4]. If the volume of the injection is more than 30 ml, the solution can extend cranially, and expand around the dural sac resulted increasing the proportion of the spinal nerve sink under the local anesthetic drug. However, CEB requires a certain volume of local anesthetic to produce a good anesthetic effect. A low volume of local anesthetic requires a large concentration to produce a good anesthetic effect. In our study, we found that the lower the volume of ropivacaine was, the greater the concentration of ropivacaine.

There are several methods that can improve the success rates of CEB. Firstly, using ultrasound-guided in-plane puncture technique, which helped me see the needle's entire length in the sacral lumen. The serious complications of caudal epidurals are accidental intrathecal injection and intravascular injection caused by variable filum terminale termination. It has been suggested that the needle should not be inserted too cephalad into the sacral canal and its tip should be visible under an ultrasound image to avoid dural puncture. Secondly, When we are not sure whether the puncture needle is in the sacral lumen for some patients with unclear anatomy, the needle tip was confirmed using a transverse view (frog’s face), and few milliliters of drug was injected in a pulsatile manner, and the expansion of the epidural space was observed.

The limitation of study is that all blocks were performed by a highly experienced anesthesiologist, which might limit the applicability of our findings. Another limitation of our study was the lack of quantitative and objective markers as indicators of a successful CEB. For example, to date, no objective measure of pain has been developed to quantify the degree of pain, and there is no way to objectively gauge how much the anal sphincter relaxes. Furthermore, sex differences have not been taken into consideration, and the guiding principle for recruiting participants was to have a mix of included and fewer excluded participants, rather than focusing on sex differences. To strengthen the validity of our study results, we need to conduct further dose-finding studies using other volumes of ropivacaine, other LAs, and out-of-plane injection techniques. Future clinical studies should focus on the optimal concentration and volume of ropivacaine for CEB and the optimum weight ratio of local anesthetic concentration and volume for CEB with the greatest benefit for anorectal surgery and minimal risk of toxicity.

## Conclusion

In summary, for ultrasound-guided CEB, a 20 ml dose of 0.362% ropivacaine or a 25 ml dose of 0.316% ropivacaine will provide adequate surgical anesthesia to 95% of patients.

## Data Availability

The datasets generated and/or analysed during the current study are not publicly available due to institutional restrictions but are available from the corresponding author on reasonable request. The email address of the corresponding author is 1197058@zju.edu.cn.
